# Dopamine-dependent oscillations in frontal cortex index “start-gun” signal in interval timing

**DOI:** 10.3389/fnhum.2015.00331

**Published:** 2015-06-12

**Authors:** Tadeusz W. Kononowicz

**Affiliations:** Cognitive Neuroimaging Unit, Commissariat Energie Atomique, DSV/I2BM, NeuroSpin, Institut National de la Santé et de la Recherche Médicale, U992, University of Paris-SudGif/Yvette, France

**Keywords:** dopamine, striatal beat frequency model, oscillatory power, interval timing, frontal cortex

Although perceiving the passage of time is a basic building block of cognitive processes and behavior such as expecting relevant events to happen, the neural underpinnings of interval timing are not well-understood as yet (van Wassenhove, 2009; Allman and Meck, 2012; Merchant et al., 2013). From the neurobiological point of view, it has been established that dopamine impacts interval timing (e.g., Meck, 1986, 1996; Allman and Meck, 2012). However, a link between pharmacological manipulations and their impact on neurophysiological signals has been rarely investigated. The leading neurobiologically plausible model of interval timing that considers both components is the Striatal Beat Frequency (SBF) model (Mattel and Meck, 2004; Buhusi and Meck, 2005; van Rijn et al., 2014). The SBF model relies on the neuromodulatory dynamics of the thalamo-cortico-striatal loops. Although currently most of the interactions in the SBF model are assumed to be unidirectional, Mattel and Meck (2004) also acknowledged the possibility of feedback from the cortex to the neurons in the VTA as well as from striatal neurons to both the cortex and the substantia nigra pars compacta. These potential feedback mechanisms are unaddressed in the SBF model. However, their implementation would allow for more accurate description of clock speed and memory updating mechanisms on a trial to trial basis (W. Meck, personal communication, May 15, 2015). Nevertheless, the SBF assumes that time is coded by the coincidental activation of striatal spiny neurons with cortical oscillators (CO). Numerical implementations of the SBF model utilizes the phase, or amplitude, of the CO that are envisioned to oscillate at various frequencies giving rise to different amplitude patterns over time as illustrated in Figure 1. Hence, at a given time point a specific amplitude pattern of the CO can be encoded by striatal spiny neurons. Crucially, the SBF model assumes that, at the onset of the to-be-timed interval, the phases of CO are reset by a burst of dopaminergic input from the ventral tegmental area (VTA, Mattel and Meck, 2004). Further, the SBF model contends that the initial dopamine-triggered phase-resetting of CO by VTA plays the role of a “start-gun” that initiates timing. This “start-gun” signal forces a whole set of CO to start from the same phase, allowing for coincidence detection to read the state of CO, and code for a particular duration over multiple trials. Importantly, the more accurate the phase-reset at the onset of the to-be-timed interval—that is, the proportion of CO that is reset to the same phase—the larger the phase synchronization and the oscillatory power of ongoing oscillations (Canavier, 2015), and as such reduces variability in memory representation of to-be-timed interval (see Figure 1; Ng et al., 2011). Within this framework more precise phase reset should be associated with an increase in timing accuracy. Note that timing accuracy can be seen as a peak latency of a response distribution, or kurtosis of a response distribution. According to the SBF model the peak latency and kurtosis of response distribution can be accounted for by different features of the model. The peak latency is modulated by frequency range of CO whereas kurtosis is accounted for by accuracy of initial phase reset (Oprisan and Buhusi, 2014). As such the width shows consistency of memory representation estimated over a number of trials. What is referred to here is the accuracy as the width of the response distribution that is associated to initial reset in terms of the SBF model. Therefore, the width of the response distribution should be affected by consistency of phase reset. However, such covariation between the precision of the “start-gun” represented by the modulation of oscillatory synchrony in any neural population and timing accuracy has not yet been tested directly.

**Figure 1:**
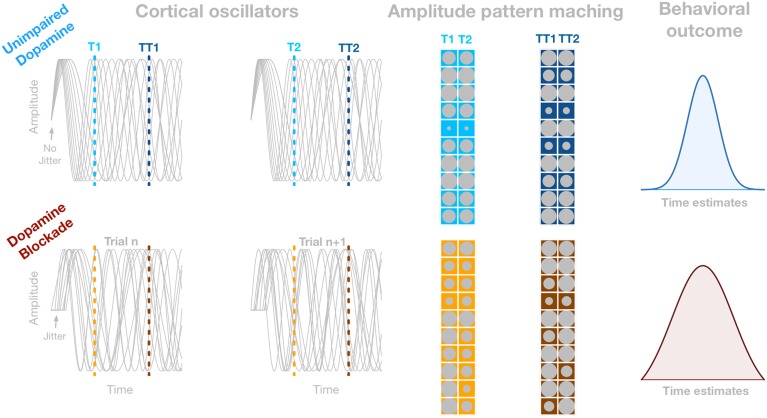
**Illustration of how dopamine-driven reduction of the precision of the “start-gun” may influence accuracy of interval timing.** The gray sinusoids in four panels depict oscillators in the theta range in two example trials (the first two columns). The first row depicts oscillatory process when dopaminergic receptors are not impaired. In this case the oscillators are perfectly synchronized. The second row depicts oscillators in the condition with impaired dopamine receptors. In this condition the onset of the oscillators are jittered, corresponding to the less precise “start-gun” mechanism. The amplitude of each oscillator is represented by the size of gray circle. Columns of matrices represent *n*, and *n* + 1 trial, respectively. Both matrices in each row depict the amplitude pattern at 200, and 450 ms that correspond to the dotted lines in the panels showing the oscillators. The amplitude/phase pattern between trials (columns of each matrix) is more dissimilar in the second row, because of the larger variability in the onset of the oscillators. Thus, if a particular amplitude/phase pattern has to be detected, the detection process will be more variable, causing larger variability in the state of oscillators around the criterion time. The right column depicts the spread of time estimations caused by jitter in the reset latency of ongoing oscillatory process.

In a recent issue of The Journal of Neuroscience, Parker et al. (2014) precisely tackled this very issue by investigating the role of dopamine-driven theta oscillations (3–8 Hz) in rodents' Medial Frontal Cortex (MFC) triggered by interval onset. Parker et al. (2014) trained rats to perform a 12 s fixed-interval timing task in which only responses longer than 12 s were reinforced. Response time was defined as the average time the rats pressed the leaver on each trial. The interval onset was signaled by turning on the house light. Importantly, after the training on interval timing task, rats were implanted with a cannula and microwire array, which allowed for the manipulation of D1 receptors and performing electrophysiological recordings. Parker et al. (2014) focused on two types of trials. In control trials the rats received saline injection whereas in the D1 blockade condition, they received D1 receptor antagonist into the MFC.

Firstly, the time-frequency analysis of local field potentials showed that the interval onset was accompanied by a significant burst of theta power as compared to baseline, indicating synchronization of neuronal populations in the MFC as proposed by the SBF model. The authors also found a decrease in beta power (15–30 Hz) at the interval onset which is not predicted by the SBF. However, the fluctuations in beta power, which are likely driven by dopamine levels in cortico-striatal circuits, may reflect mechanisms involved in interval timing (Kononowicz and van Rijn, 2014b; also see Bartolo et al., 2014; Bartolo and Merchant, 2015) that awaits future integration with the SBF model. The behavioral results showed that interval timing performance was impaired by means of blocking dopamine receptor in the D1 blockade condition compared to the control condition. The authors used the curvature index that measures the deviation from the cumulative sum of uniform response distribution, indicating kurtosis of a given distribution. Importantly, the slope of response distribution in the control condition was steeper than in the D1 blockade condition as evidenced by the difference in the curvature index. This difference indexes better timing accuracy in the control condition in which dopamine receptors were not impaired and interval onset was accompanied by the burst of theta power. Importantly, in line with the SBF model, the difference in accuracy for interval timing was accompanied by dopamine-driven attenuation of theta oscillatory power in the D1 blockade condition, showing that dopamine blockade reduces precision of the “start-gun.”

There are some arguments worth considering in conjunction with the proposed interpretation of the theta effects. Firstly, as theta modulation is typically associated with memory (Ward, 2003), the observed theta effect is unlikely to simply be caused by impaired attention, since, if this was the case, the interval onset firing rate should differ between experimental conditions (Reynolds et al., 1999). However, no such pattern was reported.

Parker et al. (2014) also analyzed the role of oscillatory power (1–12 Hz, ranging from 0 to 6 s after interval onset) and climbing neural activity (CNA) that has been proposed to code for subjective time (for review, see Merchant et al., 2013). Importantly, although partial correlation analysis revealed that response times were more powerfully associated with CNA than with 1–12 Hz oscillatory power, this result does not preclude the main proposal that dopamine-driven theta oscillations index the “start-gun” mechanism. In other words, since partial correlation analysis focused on the response time latency, it did not take into account the shape of overall response distribution—a key result in support of the “start-gun hypothesis.” As shape and peak latency of response distribution could arise from different features of the SBF model, future studies should consider investigating both of these elements in order to shed more light on the interdependency of the “start-gun,” and the frequency of oscillators, their relation to different types of dopaminergic receptors, and phasic and tonic dopamine levels (Cohen et al., 2002).

The next consideration relates to the range of oscillatory power that was analyzed. Note that although Parker et al. (2014) acknowledge the role of 3–8 Hz oscillations in interval timing, the authors put a bigger emphasis on the role of CNA since it was correlated more strongly with response times than the 1–12 Hz oscillatory power, ranging from 0 to 6 s after interval onset. However, analysis targeting more specific component (e.g., 3–8 Hz power ranging from 0 to 1 s, identified in the earlier section of the paper), linked here to the “start-gun” signal, may instead emphasize the theta oscillatory mechanism that would lend further support for the SBF model. Additionally, contrary to the conclusion by Parker et al. (2014), climbing activity has recently been questioned as a key mechanism of interval timing (Kononowicz and van Rijn, 2011; Ng et al., 2011; van Rijn et al., 2011; Mento et al., 2013; Kononowicz et al., 2015; Wiener and Thompson, 2015; but see Wiener et al., 2012; Herbst et al., 2014). For example, Kononowicz and van Rijn (2014a) showed that auditory component demarcating the end of an interval was a better measure of the interval than CNA instantiated by contingent negative variation recorded from the human scalp. Despite these differences both studies point toward the novel neural markers of interval timing associated with the onset and offset of to-be-timed interval.

Interestingly, the theta effects reported by Parker et al. (2014) also have other implications for the SBF model in that it suggests a possible range of CO utilized for coincidence detection. This frequency range is lower than the alpha oscillations (8–12 Hz) typically associated with the frequency range modeled by CO in the SBF model (Oprisan and Buhusi, 2014), and were previously conceived as a marker of the internal clock (Treisman, 1984). However, unlike previous reports, the study by Parker et al. (2014) demonstrated that the spatial location of theta effects is congruent with the neural structures implicated in the SBF model by showing that neuronal assemblies in the MFC reset upon the occurrence of the to-be-timed interval. Moreover, similarly to alpha oscillations, theta oscillations have also been suggested as playing an important role in working memory (Jensen and Tesche, 2002; Gulbinaite et al., 2014). Interestingly, a recent review by Gu et al. (2015) proposes a way of integrating interval timing and working memory into one model in which theta oscillations serve as a neural code indexing temporal order, and duration information (Kösem et al., 2014).

In conclusion, the study by Parker et al. (2014) provides the first evidence that phase reset of theta oscillations at the interval onset influences timing accuracy. Thus it strengthens the SBF model and provides insights for future experimental and modeling work unraveling neural bases of interval timing.

## Conflict of interest statement

The author declares that the research was conducted in the absence of any commercial or financial relationships that could be construed as a potential conflict of interest.

## References

[B1] Allman M. J. Meck W. H. (2012). Pathophysiological distortions in time perception and timed performance. Brain 135, 656–677. 10.1093/brain/awr210 21921020 PMC3491636

[B2] Bartolo R. Merchant H. (2015). β oscillations are linked to the initiation of sensory-cued movement sequences and the internal guidance of regular tapping in the monkey. J. Neurosci. 35, 4635–4640. 10.1523/JNEUROSCI.4570-14.2015 25788680 PMC6605135

[B3] Bartolo R. Prado L. Merchant H. (2014). Information processing in the primate basal ganglia during sensory-guided and internally driven rhythmic tapping. J. Neurosci. 34, 3910–3923. 10.1523/jneurosci.2679-13.2014 24623769 PMC6705277

[B4] Buhusi C. V. Meck W. H. (2005). What makes us tick? Functional and neural mechanisms of interval timing. Nat. Rev. Neurosci. 6, 755–765. 10.1038/nrn1764 16163383

[B5] Canavier C. C. (2015). Phase-resetting as a tool of information transmission. Curr. Opin. Neurobiol. 31, 206–213. 10.1016/j.conb.2014.12.003 25529003 PMC4375052

[B6] Cohen J. D. Braver T. S. Brown J. W. (2002). Computational perspectives on dopamine function in prefrontal cortex. Curr. Opin. Neurobiol. 12, 223–229. 10.1016/S0959-4388(02)00314-8 12015241

[B7] Gu B. M. van Rijn H. Meck W. H. (2015). Oscillatory multiplexing of neural population codes for interval timing and working memory. Neurosci. Behav. Rev. 48, 160–185. 10.1016/j.neubiorev.2014.10.008 25454354

[B21] Gulbinaite R. van Rijn H. Cohen M. X. (2014). Fronto-parietal network oscillations reveal relationship between working memory capacity and cognitive control. Front. Hum. Neurosci. 8. 25324759 10.3389/fnhum.2014.00761 PMC4179713

[B8] Herbst S. K. Chaumon M. Penney T. B. Busch N. A. (2014). Flicker-induced time dilation does not modulate EEG correlates of temporal encoding. Brain Topogr. 1–11. 10.1007/s10548-014-0389-z 25117576

[B21a] Jensen O. Tesche C. D. (2002). Frontal theta activity in humans increases with memory load in a working memory task. Eur. J. Neurosci. 15, 1395–1399. 11994134 10.1046/j.1460-9568.2002.01975.x

[B9] Kononowicz T. W. van Rijn H. (2011). Slow potentials in time estimation: the role of temporal accumulation and habituation. Front. Integr. Neurosci. 5:48. 10.3389/fnint.2011.00048 21949505 PMC3171873

[B10] Kononowicz T. W. van Rijn H. (2014a). Decoupling interval timing and climbing neural activity: a dissociation between CNV and N1P2 amplitudes. J. Neurosci. 34, 2931–2939. 10.1523/JNEUROSCI.2523-13.2014 24553934 PMC6608524

[B11] Kononowicz T. W. van Rijn H. (2014b). Tonic and phasic dopamine fluctuations as reflected in beta power predict interval timing behavior. Procedia Soc. Behav. Sci. 126, 47 10.1016/j.sbspro.2014.02.313

[B11a] Kononowicz T. W. Sander T. Van Rijn H. (2015). Neuroelectromagnetic signatures of the reproduction of supra-second durations. Neuropsychologia 10.1016/j.neuropsychologia.2015.06.001 26057434

[B12] Kösem A. Gramfort A. van Wassenhove V. (2014). Encoding of event timing in the phase of neural oscillations. Neuroimage 92, 274–284. 10.1016/j.neuroimage.2014.02.010 24531044

[B13] Mattel M. S. Meck W. H. (2004). Cortico-striatal circuits and interval timing: coincidence detection of oscillatory processes. Brain Res. Cogn. Brain Res. 21, 139–170. 10.1016/j.cogbrainres.2004.06.012 15464348

[B14] Meck W. H. (1986). Affinity for the dopamine D 2 receptor predicts neuroleptic potency in decreasing the speed of an internal clock. Pharmacol. Biochem. Behav. 25, 1185–1189. 10.1016/0091-3057(86)90109-7 2880350

[B15] Meck W. H. (1996). Neuropharmacology of timing and time perception. Cogn. Brain Res. 3, 227–242. 10.1016/0926-6410(96)00009-2 8806025

[B16] Mento G. Tarantino V. Sarlo M. Bisiacchi P. S. (2013). Automatic temporal expectancy: a high-density event-related potential study. PLoS ONE 8:e62896. 10.1371/journal.pone.0062896 23650537 PMC3641105

[B17] Merchant H. Harrington D. L. Meck W. H. (2013). Neural basis of the perception and estimation of time. Annu. Rev. Neurosci. 36, 313–336. 10.1146/annurev-neuro-062012-170349 23725000

[B18] Ng K. K. Tobin S. Penney T. B. (2011). Temporal accumulation and decision processes in the duration bisection task revealed by contingent negative variation. Front. Integr. Neurosci. 5:77. 10.3389/fnint.2011.00077 22144952 PMC3225905

[B19] Oprisan S. A. Buhusi S. V. (2014). What is all the noise about in interval timing? Philos. Trans. R. Soc. B Biol. Sci. 369, 20120459. 10.1098/rstb.2012.0459 24446493 PMC3895984

[B20] Parker K. L. Chen K. H. Kingyon J. R. Cavanagh J. F. Narayanan N. S. (2014). D_1_-dependent 4 Hz oscillations and ramping activity in rodent medial frontal cortex during interval timing. J. Neurosci. 34, 16774–16783. 10.1523/JNEUROSCI.2772-14.2014 25505330 PMC4261101

[B22] Reynolds J. H. Chelazzi L. Desimone R. (1999). Competitive mechanisms subserve attention in macaque areas V2 and V4. J. Neurosci. 19, 1736–1753. 10024360 10.1523/JNEUROSCI.19-05-01736.1999 PMC6782185

[B23] Treisman M. (1984). Temporal rhythms and cerebral rhytms. Ann. N.Y. Acad. Sci. 423, 542–565. 10.1111/j.1749-6632.1984.tb23458.x 6588814

[B24] van Rijn H. Gu B.-M. Meck W. H. (2014). Dedicated clock/timing-circuit theories of interval timing and timed behavior. Adv. Exp. Med. Biol. 829, 75–99. 10.1007/978-1-4939-1782-2_5 25358706

[B25] van Rijn H. Kononowicz T. W. Meck W. H. Ng K. K. Penney T. B. (2011). Contingent negative variation and its relation to time estimation: a theoretical evaluation. Front. Integr. Neurosci. 5:91. 10.3389/fnint.2011.00091 22207841 PMC3246349

[B26] van Wassenhove V. (2009). Minding time in an amodal representational space. Philos. Trans. R. Soc. B Biol. Sci. 364, 1815–1830. 10.1098/rstb.2009.0023 19487185 PMC2685822

[B27] Ward L. M. (2003). Synchronous neural oscillations and cognitive processes. Trends Cogn. Sci. 7, 553–559. 10.1016/j.tics.2003.10.012 14643372

[B28] Wiener M. Kliot D. Turkeltaub P. E. Hamilton R. H. Wolk D. A. Coslett H. B. (2012). Parietal influence on temporal encoding indexed by simultaneous transcranial magnetic stimulation and electroencephalography. J. Neurosci. 32, 12258–12267. 10.1523/JNEUROSCI.2511-12.2012 22933807 PMC3448365

[B29] Wiener M. Thompson J. C. (2015). Repetition enhancement and memory effects for duration. Neuroimage 113, 268–278. 10.1016/j.neuroimage.2015.03.054 25818689

